# Practical Exploration of Medical Students' Curriculum Education Model in the Context of New Medical Science—Taking Biochemistry and Molecular Biology as an Example

**DOI:** 10.1002/bmb.70019

**Published:** 2025-10-27

**Authors:** Shuzi Xu, Yiyang Chen, Juanjuan Wu

**Affiliations:** ^1^ Nantong University Nantong Jiangsu China

**Keywords:** biochemistry and molecular biology, educational reform, medical students, new medical science

## Abstract

This study evaluates a reformed teaching model for biochemistry and molecular biology under New Medical Science, comparing traditional methods with a blend of online–offline learning, flipped classrooms, and humanistic integration. A quasi‐experiment with 351 medical students showed that the experimental group (stomatology) outperformed the control (clinical medicine) in exam scores: 2020–2022 cohorts had mean score increases of 2.76–3.92 (all *p* < 0.05). Satisfaction surveys indicated 93.2% approved the evaluation mechanism, 94.9% linked biochemistry cases to ethical reflection, and 64.4% reported heightened research motivation. The model enhances academic performance and cultivates comprehensive competencies, meeting modern medical education needs.

## 1 Introduction

New Medical Science (NMS) is a key initiative in China's higher medical education reform, which emphasizes the integration of medical education with technological innovation (e.g., artificial intelligence, precision medicine) and humanistic ethics to cultivate versatile healthcare professionals capable of addressing complex healthcare challenges [1]. Specifically, it advocates breaking disciplinary barriers, strengthening the connection between basic medical sciences and clinical practice, and highlighting the cultivation of both professional skills and humanistic literacy [2]. In the context of this study, NMS provides a directional guide for the reform of biochemistry and molecular biology courses, requiring basic medical education to break through the limitations of traditional knowledge transmission [3] and realize the integration of disciplinary knowledge, technical application, and humanistic literacy, which is exactly the core goal of this teaching reform practice. Under this framework, biochemistry and molecular biology education faces the challenge of transcending traditional knowledge transmission to foster comprehensive competencies—including critical thinking, ethical reasoning, and interdisciplinary application [3, 4].

To address this, the reformed model integrates three key strategies: online–offline blended learning, flipped classroom instruction, and case‐based humanistic‐ethical integration [5, 6, 7]. This approach aims to organically combine knowledge education, ability cultivation, and quality development, aligning with NMS demands for innovative medical training.

Against this backdrop, this study adopts a quasi‐experimental design involving 351 medical students at Nantong University to evaluate a reformed model integrating online–offline blended learning, flipped classrooms, and humanistic‐ethical integration—strategies tailored to NMS's demands. Specifically, we aimed to explore: (1) whether these adapted methods enhance academic performance in biochemistry and molecular biology, a foundational discipline for NMS competencies; (2) whether they effectively cultivate critical thinking, ethical reasoning, and interdisciplinary application abilities required by NMS. By analyzing academic performance (Tables 1, 2, 3) and competency development (Table 4), this study demonstrates how targeted pedagogical reforms align with modern medical education needs, providing empirical support for NMS‐driven curriculum innovation.

**TABLE 1:** Legend for the independent sample *t*‐test results table of grades of two majors in the class of 2020.

	Specialized field	*N*	Average value	(statistics) Standard deviation	*t*	*p*
Grades	Stomatology	60	75.58	8.156	2.105	0.037
	Preclinical	60	72.82	6.097		

**TABLE 2:** Legend for the independent sample *t*‐test results table of grades of two majors in the class of 2021.

	Specialized field	*N*	Average value	(statistics) Standard deviation	*t*	*p*
Grades	Stomatology	55	76.71	9.895	2.347	0.021
	Preclinical	56	72.91	6.855		

**TABLE 3:** Legend for the Independent Sample t—Test Results Table of Grades of Two Majors in the Class of 2022.

	Specialized field	*N*	Average value	(statistics) Standard deviation	*t*	*p*
Grades	Stomatology	60	79.07	10.412	2.088	0.039
	Preclinical	60	75.15	10.131		

**TABLE 4:** Satisfaction of students (*n* = 59) with the reformed biochemistry program.

Questionnaire	Results of investigation
By previewing this course online, you will be able to better assess your knowledge, resolve your questions, and improve your listening efficiency when listening to the lectures offline. [single choice]	Strongly disagree (0%); disagree (0%); neutral (11.86%); agree (28.81%); strongly agree (59.32%)
When confronted with a new idea, your ability to explore the truth with a critical eye through a variety of resourceful avenues helps to enhance your problem‐solving skills. [single choice]	Strongly disagree (0%); disagree (0%); neutral (8.47%); agree (35.59%); strongly agree (55.93%)
This course focuses on group cooperation and the exchange of different opinions. It helps develop listening skills and conflict resolution, enhancing interpersonal skills. [single choice]	Strongly disagree (0%); disagree (0%); neutral (6.78%); agree (32.2%); strongly agree (61.02%)
When assigned a research direction, you will review existing literature to expand your thinking and identify a new research topic. [single choice]	Strongly disagree (0%); disagree (0%); neutral (6.78%); agree (30.51%); strongly agree (62.71%)
This course's learning process incorporates stories of Nobel Laureates in physiology or medicine, helping to stimulate your interest in scientific research and innovative thinking. [single choice]	Strongly disagree (0%); disagree (0%); neutral (5.08%); agree (30.51%); strongly agree (64.41%)
In which of the following areas did you have an improvement in your skills or abilities as a result of this course? [multiple choice questions]	Literature reading (71.19%); teamwork and collaboration (74.58%); PPT production (71.19%); presentation skills (61.02%); creative thinking (59.32%)

## 2 Dilemmas in Traditional Classroom Teaching of Biochemistry

In the context of New Medical Science (NMS), biochemistry and molecular biology education must transcend traditional knowledge transmission to align with NMS goals: integrating technical innovation (e.g., precision medicine) and fostering competencies like critical thinking, ethical reasoning, and clinical translation [8]. This requires shifting from passive knowledge delivery to cultivating versatile skills for complex healthcare challenges.

Traditional teaching, while foundational, struggles to meet these NMS demands. Its over‐reliance on teacher‐centered instruction and summative assessment (e.g., final exams as primary evaluation) limits opportunities for clinical application, interdisciplinary connection, and ethical reflection—key NMS competencies [9, 10]. Thus, adapting proven pedagogies to NMS‐specific objectives becomes critical to address this gap.

## 3 Basic Strategies of the Biochemical Rehabilitation Curriculum Model

**FIGURE 1:**
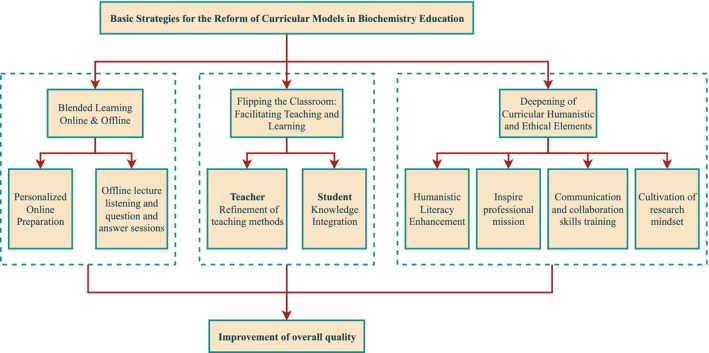
Basic strategies for the reform of curricular models in biochemistry Education.

Aligned with the core goals of New Medical Science (NMS)—integrating technological innovation, clinical translation, and humanistic ethics—this reform adopts three targeted strategies to cultivate versatile medical professionals [11, 12]. These strategies are not merely adaptations of existing methods but are tailored to NMS's demand for “knowledge application + competency development + value shaping” (Figure 1).

### 3.1 Blended Learning: Online and Offline

To address NMS's emphasis on “technology‐medical integration,” blended learning is designed to connect basic biochemistry with cutting‐edge medical practice. Pre‐class online modules (e.g., MOOCs on gene editing mechanisms) enable students to master foundational knowledge independently [13, 14], while in‐class offline sessions focus on applying this knowledge to clinical scenarios (e.g., discussing CRISPR technology in precision medicine). This structure not only optimizes lecture efficiency but also directly bridges disciplinary gaps—key to NMS's interdisciplinary training objectives.

### 3.2 Flipped Classroom Teaching and Learning

To respond to NMS's demand for cultivating “problem‐solvers” rather than “knowledge receivers,” the flipped classroom model was adapted to emphasize clinical‐translational thinking. The model retains its core design where students independently acquire knowledge through pre‐class online materials (e.g., biochemical pathways like the Krebs cycle), then engage in active in‐class interactions. A key feature is that students take on the role of “little teachers”: they take turns presenting key concepts (e.g., explaining metabolic regulation mechanisms to peers) and leading discussions, which shifts the traditional teacher‐centered mode to peer‐driven learning. This “little teacher” approach not only enhances students' mastery of knowledge through teaching others but also develops their communication and leadership skills—critical for NMS's goal of training collaborative healthcare professionals.

Teachers, as “coaches,” observe these presentations to identify knowledge gaps (e.g., misunderstandings of enzyme kinetics) and provide targeted guidance [15]. For example, if students struggle to link glycolysis to clinical hypoglycemia, teachers guide discussions on translating biochemical principles into diagnostic reasoning. Additionally, instructors design questions to help students connect prior and subsequent knowledge (e.g., linking cellular respiration to cancer metabolism), constructing a knowledge network that supports clinical application. This structure not only strengthens student‐teacher interaction but also fosters the analogy, comparison, and dialectical thinking required for complex healthcare challenges—directly aligning with NMS's competency demands.

### 3.3 Deepening of Curricular Humanistic and Ethical Elements

In the context of new medical science, a core objective of medical education is to enhance students' humanistic and ethical literacy, including professional ethics, social responsibility, and patient‐centered care. Medical students require qualities beyond medical knowledge, such as lifelong humanistic values and empathy for patients, which are nurtured through integrated curriculum design [16, 17].

The biochemistry reform model incorporates humanistic education via small‐group case analyses—retaining the essence of linking scientific content to ethical reasoning. For instance, when exploring viral‐host interactions in COVID‐19, students not only dissect scientific literature on viral protein structures but also analyze dilemmas such as resource allocation during pandemics and the ethical balance between research urgency and patient safety [18, 19]. This design prompts reflection on the professional dedication of medical workers, fostering appreciation for ethical decision‐making in public health crises. Additionally, examining the research journeys of Nobel laureates in physiology or medicine—such as Alexander Fleming's patient‐centered approach during penicillin trials—illuminates the moral foundations of scientific inquiry [20]. These cases are intentionally aligned with NMS's emphasis on “ethical practice in clinical and research settings,” systematically cultivating students' professional ethics and critical thinking [21].

In the Civics course, participants engage in a reciprocal learning environment where they gain knowledge from one another, assess and balance one another's areas of improvement and deficit, expand their perspectives, foster communication and collaboration, plan their studies, and create PowerPoint presentations for on‐stage presentations [22]. Additionally, open‐ended learning activities, which are integrated into the course, could promote students' rationality, decision‐making, problem‐solving, and negotiation skills in their own lives [23].

## 4 Materials and Methods

### 4.1 Experimental Design

We utilized descriptive qualitative research and a combined research methodology, which included a quasi‐experimental design, to establish the traditional teaching group and the teaching reform group.

### 4.2 Subjects of the Study

The survey participants comprised 351 first‐year students enrolled in the 2020–2022 academic years at Nantong University School of Medicine, with concentrations in stomatology and clinical medicine. The inclusion criteria for participants were as follows: (1) absence of current abnormal physical or mental symptoms; (2) enrolling in full‐time medical undergraduate programs; (3) no prior experience in teaching and reforming courses in medical specialties; (4) absence of a statistically significant difference in learning level and ability between the two classes prior to the experiment; and (5) completion of an informed consent form to voluntarily partake in this study. The control group comprised 60 students from the 2020 clinical medicine program, 56 students from the 2021 clinical medicine program, and 60 students from the 2022 clinical medicine program, respectively, and utilized the traditional teaching mode. Sixty students from the 2020 stomatology program, 62 students from the 2021 stomatology program, and 60 students from the 2022 stomatology program comprised the experimental group, which implemented the new teaching mode of reform within the framework of new medicine.

For a total of 12 years of education, all students were admitted directly to the university via the National High School Examination during their senior year of high school. No statistically significant disparities were observed between the control and experimental groups of students in terms of age, enrollment, grades, or the male‐to‐female ratio. During their first semester of their second year of college, they studied molecular biology and biochemistry. The faculty members were comparable, and both student groups utilized the ninth edition of [24].

### 4.3 Research Methodology

All methods were executed in accordance with the pertinent protocols. Instructors from the Biochemistry Teaching and Research Department of Nantong University taught both the experimental and control groups using the traditional and new methods of reform education, respectively. The teaching mode utilized for the clinical medicine specialty from 2020 to 2022 adhered to the conventional teaching mode. Specifically, instructors presented theories through multimedia means, while students engaged in textbook‐based lectures, note‐taking, and exam review. The specialization in stomatology for 2020–2022 implemented the new mode of education reform.

### 4.4 Evaluation Methodology

Assessment of student academic performance was based on final exam scores, while student satisfaction with the course (including perceptions of teaching characteristics) was evaluated through online surveys. By consulting the question repository, the teachers of the Biochemistry Teaching and Research Office at Nantong University School of Medicine selected the questions for the final examination. The test questions are derived from the course syllabus and basic requirements, emphasizing the most significant concepts and difficult aspects of the curriculum. In general, the test paper possesses a moderate level of difficulty. The examination comprised 100 single‐choice questions, the majority of which assessed students' mastery of biochemistry theory and knowledge application in their entirety. Students were provided with the satisfaction survey via a questionnaire star link.

### 4.5 Statistical Methods

The statistical software SPSS 26.0 was utilized for data processing and analysis. The t‐test was applied to measurement data expressed as (mean ± standard deviation), while the *χ* ^2^ test was utilized for count data expressed as (*n*, %). Significant statistical values.

## 5 Results

### 5.1 Comparison of Student Performance

The experimental and control groups underwent identical and comparable final exams; the following are the results:

First, a *t*‐test on independent samples was conducted on the grades of the two majors in the class of 2020. The subsequent table (Table 1) presents the results:

In the class of 2020, there were 60 students enrolled in the clinical program and 60 students enrolled in the stomatology program. The mean grades for the clinical program were 72.82 with a standard deviation of 6.10, while the mean grades for the stomatology program were 75.58 with a standard deviation of 8.16. Based on the obtained *t*‐statistic value of 2.105 and the corresponding significance level of 0.037 (less than 0.05), it can be concluded that a notable disparity existed between the grades of the clinical and stomatology programs, with the stomatology program receiving significantly higher grades.

In the table below (Table 2), the independent sample *t*‐test for the Class of 2021 grades in both majors is displayed:

The class of 2021 comprised 56 students enrolled in the clinical program and 55 students enrolled in the stomatology program. The clinical program grades had a mean value of 72.91 and a standard deviation of 6.86, while the stomatology program grades had a mean value of 76.71 with a standard deviation of 9.90. Based on the *t*‐statistic value of 2.347 and the associated significance level of 0.021 (less than 0.05), it can be concluded that the grades for the clinical and stomatology programs differ significantly, with the stomatology program's grades being significantly higher than those for the clinical program.

The table below (Table 3) displays the results of the independent sample *t*‐tests conducted on the grades of the two majors comprising the class of 2022:

In the class of 2022, there were 60 students enrolled in the clinical program and 60 students enrolled in the stomatology program. The clinical program grades had a mean value of 75.15 and a standard deviation of 10.13, whereas the stomatology program grades had a mean value of 79.07 and a standard deviation of 10.41. Based on the obtained t‐statistic value of 2.088 and the corresponding significance level of 0.039 (less than 0.05), it can be concluded that a statistically significant distinction existed between the grades of the clinical and stomatology programs, with the stomatology program receiving significantly higher grades.

### 5.2 Questionnaire Star Satisfaction Survey

**FIGURE 2:**
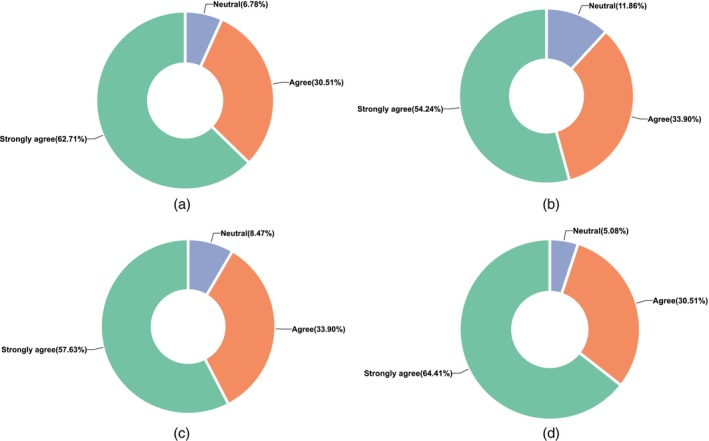
Survey via an online questionnaire. (a) The evaluation mechanism for the course is well‐designed. It enables process evaluation that precisely reflects your learning at each stage. (b) Throughout the course of the program, you have identified your optimal learning style and method, leading to a notable enhancement in your learning efficacy. (c) Teachers' questions can assist students in the flipped classroom in recognizing knowledge gaps and comprehending the relationships between concepts. This could result in an enhanced comprehension of the topic. (d) Real‐world examples from the field of biochemistry stimulate reflection on the applicability of this course to societal needs, public health, and ethics, among other pertinent domains.

For the reformed biochemistry course, an online survey was conducted among 62 invited students. After a 1‐week questionnaire process, during which cross‐analysis was carried out and invalid questionnaires were eliminated, 59 valid ones were obtained, resulting in a 95% completion rate. The findings of the survey are depicted in Figure 2.

The majority of students expressed agreement regarding the teaching effect of the course reform model (Table 4). By combining online and offline blended learning, students are able to comprehend material and solve questions more effectively, thereby maximizing the effectiveness of lecture listening. By encouraging students to develop their critical thinking, collaboration, and interpersonal skills, as well as foster innovative thinking, this mode of educational reform can assist students in becoming medical innovators and enhance their overall humanistic literacy.

The survey results validate the effectiveness of humanistic‐ethical integration: 94.92% of students agreed that biochemical cases (e.g., COVID‐19 research ethics) stimulated reflection on public health and ethics (Table 4), indicating that real‐world scenarios effectively link scientific content to ethical reasoning. Furthermore, 64.41% reported enhanced curiosity for scientific research after engaging with Nobel laureate case studies (Table 4, Question 5), confirming the value of scientific history in fostering innovative thinking. Notably, 71.19% of students showed improved literature reading skills (Table 4), a direct outcome of analyzing ethical case studies through primary research articles. These findings demonstrate that integrating humanistic elements not only enriches ethical literacy but also strengthens core academic competencies.

## 6 Discussion

### 6.1 Academic Performance Enhancement

The experimental group demonstrated significant score improvements across all cohorts, with mean scores 2.76–3.92 points higher than the control group (Tables 1, 2, 3). This correlates with 59.32% of students strongly agreeing that online pre‐learning enhanced offline listening efficiency (Table 4), indicating blended learning effectively optimizes knowledge acquisition—aligning with NMS's emphasis on active knowledge application. The flipped classroom further contributed, as 61.02% reported better presentation skills and 57.63% found teacher‐led questions helped identify knowledge gaps (Figure 2c), directly supporting deeper mastery of biochemistry concepts (e.g., enzyme regulation) critical for NMS's demand for clinical reasoning [24].

### 6.2 Effectiveness of Humanistic‐Ethical Integration

Ethical case studies showed substantial impact, with 94.92% of students agreeing that real‐world scenarios (e.g., COVID‐19 resource allocation) stimulated public health ethics reflection (Figure 2d)—directly addressing NMS's focus on ethical reasoning in healthcare practice. Notably, 64.41% reported heightened research motivation through Nobel laureate cases (Table 4), while 71.19% improved literature reading skills—skills critical for NMS's demand for evidence‐based ethical decision‐making. These findings validate that integrating ethics enhances both professional virtue and academic competency, aligning with NMS's holistic training goals.

### 6.3 Comparison With Existing Research

The results align with Nichat et al., who found flipped classrooms improve critical thinking in medical education [25]. Our study extends this by demonstrating how such methods, when tailored to NMS goals (e.g., embedding clinical ethics in case studies), enhance both academic performance and ethical literacy. Additionally, process evaluation (50% of total grade) effectively captures formative development (93.22% approval, Figure 2a), surpassing traditional models and directly responding to NMS's call for comprehensive competency assessment.

**FIGURE 3:**
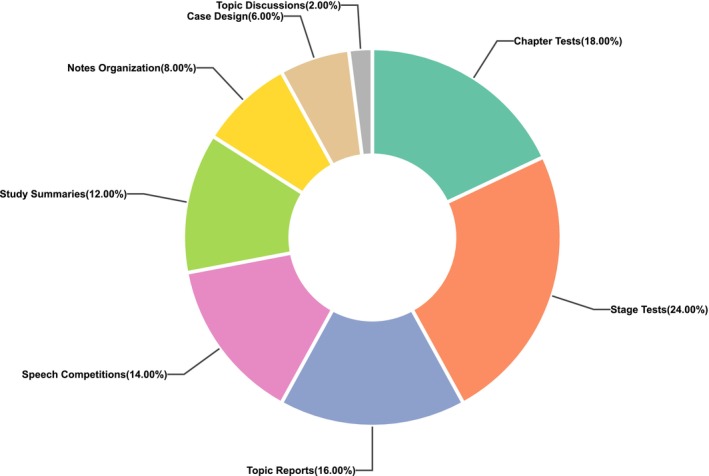
Proportion of process evaluations.

In contrast to the conventional educational model, the biochemical education reform course has augmented the proportion attributed to process evaluation with regard to the evaluation mechanism (Figure 3). The degree of achievement of the course objectives is assessed via the process evaluation (worth 50% of the total grade) and the final examination (worth 50% of the total grade), thereby guaranteeing a scientific and unbiased evaluation of the grades [26].

The process evaluation plays a crucial role in comprehensively assessing students' learning throughout the course. It is designed to cover multiple aspects of students' learning activities and skills. For example, topic discussions, which account for 2% of the total grade, are conducted via specific apps. Students express their views and interact on preset biochemical topics to enhance their understanding of knowledge. Case designs, making up 6% of the total grade, require students to create practical biochemical cases using the knowledge they have learned. These cases are judged based on creativity, the accuracy of concepts, and the feasibility of solutions. Note organizations, taking 8% of the total grade, encourage systematic note‐taking. They are evaluated based on note structure, key points, and review convenience to help students with knowledge review. Study summaries, accounting for 12% of the total grade, are regularly assigned for students to summarize learning units, including concepts, difficulties, and solutions, to strengthen their knowledge and self‐learning abilities. Speech competitions, with a 14% share of the total grade, involve students choosing biochemical topics for speeches. These are evaluated based on content quality, delivery skills, and audience engagement. Topic reports, accounting for 16% of the total grade, require students to research specific topics, review literature, analyze data, and present their findings to develop research and writing skills. Stage tests, taking 24% of the total grade, are held at different intervals during the semester with various question types to identify learning gaps and evaluate students' understanding and application of knowledge in specific stages. Chapter tests, making up 18% of the total grade, focus on chapter content and test students' memory and understanding of key elements in each chapter to ensure a solid grasp of basic knowledge. Through these components, we can evaluate students' participation levels, their ability to apply knowledge in practical situations, their capacity to summarize and reflect on their learning, as well as their presentation and research skills.

The final examination, on the other hand, also contributes 50% of the total grade. It focuses on assessing students' overall understanding and mastery of the key knowledge points covered in the entire course.

## 7 Reflections and Future Directions

### 7.1 Practical Insights From Implementation

Blended learning revealed a need for personalized support: while 59.32% benefited from online pre‐learning, 11.86% remained neutral (Table 4), indicating varied self‐study abilities. Future reforms should offer adaptive online resources, such as implementing AI‐based diagnostic tools to identify students' gaps in gene expression regulation (a common difficulty in biochemistry) and deliver targeted learning resources [27].

It should be noted, however, that learning effectiveness depends greatly on students' active learning attitude and self‐study abilities [28]. Students who are highly motivated to learn, capable of learning independently, and willing to actively express themselves will benefit more from the flipped classroom model. Conversely, teachers must provide more assistance and guidance to students who are less motivated to learn, lack self‐control, and lack confidence in their ability to learn in order to optimize the benefits for a greater number of students.

### 7.2 Limitations and Future Plans

The single‐institution sample limits generalizability. Multicenter trials are needed to validate the model's efficacy across diverse student populations [29]. Additionally, the 64.41% approval rate for Nobel laureate cases (Table 4) suggests adding Chinese medical ethics cases, such as the 2020 COVID‐19 research ethics guidelines, to enhance cultural relevance [30].

### 7.3 Innovations for Continuous Improvement

To deepen ethical integration, we propose “ethical dilemma simulation” modules on topics like genetic testing confidentiality, building on the 94.92% approval of real‐world cases (Figure 2d) [31]. Combining this with expanded process evaluation components (e.g., adding clinical case analysis to the 16% topic report requirement) will further align education with modern healthcare demands [32].

Overall, against the backdrop of the development of new medical science, we adopt a series of effective approaches in the medical student curriculum education model, including online and offline blended learning, flipped classroom teaching and learning, as well as integration of humanistic and ethical elements. These strategies aim to foster medical students' professional competence, ethical awareness, and cross‐disciplinary skills, rather than ideological indoctrination. By emphasizing evidence‐based practice and patient‐centered care, the model equips students to address complex healthcare challenges—aligning with global medical education standards for holistic professional development.

## Ethics Statement

This study was performed in accordance with the Helsinki Declaration and was approved by the Ethics committee of Nantong University. The informed consent obtained from study participants was written.

## Conflicts of Interest

The authors declare no conflicts of interest.

## Data Availability

The data that support the findings of this study are available from the corresponding author upon reasonable request.
